# Novel Antibacterial Activity of β_2_-Microglobulin in Human Amniotic Fluid

**DOI:** 10.1371/journal.pone.0047642

**Published:** 2012-11-07

**Authors:** Jin-Young Kim, Seong-Cheol Park, Jong-Kook Lee, Sang Joon Choi, Kyung-Soo Hahm, Yoonkyung Park

**Affiliations:** 1 Research Center for Proteinaceous Materials, Chosun University, Kwangju, Korea; 2 Department of Obstetrics and Gynecology, School of Medicine, Chosun University, Kwangju, Korea; 3 Bioleaders Corporation, Yusong-Ku, Daejeon, Korea; 4 Department of Biotechnology, Chosun University, Kwangju, Korea; Swedish University of Agricultural Sciences, Sweden

## Abstract

An antibacterial protein (about 12 kDa) was isolated from human amniotic fluid through dialysis, ultrafiltration and C18 reversed-phase HPLC steps. Automated Edman degradation showed that the N-terminal sequence of the antibacterial protein was NH_2_-Ile-Gln-Arg-Thr-Pro-Lys-Ile-Gln-Val-Tyr-Ser-Arg-His-Pro-Ala-Glu-Asn-Gly-. The N-terminal sequence of the antibacterial protein was found to be identical to that of β_2_-microglobulin, a component of MHC class I molecules, which are present on all nucleated cells. Matrix-assisted laser desorption ionization mass spectrometry (MALDI-MS) revealed that the molecular mass of the antibacterial protein was 11,631 Da. This antibacterial protein, β_2_M, possessed potent antibacterial activity against pathogenic bacteria. Specially, antibacterial activity was observed in potassium buffer, and potassium ion was found to be critical for the antibacterial activity. Interestingly, the antibacterial action of β_2_M was associated with dissipation of the transmembrane potential, but the protein did not cause damage to the membrane that would result in SYTOX green uptake. In addition, stimulation of WISH amniotic epithelial cells with the bacterial endotoxin lipopolysaccharide (LPS) induced dose-dependent upregulation of β_2_M mRNA expression. These results suggest that β_2_M contributes to a self-defense response when amniotic cells are exposed to pathogens.

## Introduction

In humans, antimicrobial peptides and proteins are the first line of defense against bacteria, fungi and enveloped viruses [1], [2]. Amniotic fluid was recently reported to have antimicrobial properties [3], [4], and several antimicrobial peptides and proteins, including human neutrophil peptides 1, 2 and 3 [5], lysozyme [6], bactericidal/permeability-increasing protein (BPI) [7], LL-37 [6], calprotectin (MRP8/14) [8] and ubiquitin [9], have since been found in amniotic components. β_2_-microglobulin, which was isolated from human amniotic fluid (HAF) in the present study, is the noncovalently bound light chain of major histocompatibility (MHC) class I, an 11.6 kDa nonglycosylated protein found on the surface of all nucleated cells [10]. MHC class I molecules play an important role in alerting the immune system to the presence of virally infected cells. β_2_-microglobulin is an interesting and underutilized metabolite that can be used to assess renal function, particularly in kidney-transplant recipients and in patients suspected of having renal tubulo-interstitial disease [11]. It can also serve as a nonspecific but relatively sensitive marker for various neoplastic, inflammatory and infectious conditions [12]. Early hopes that β_2_-microglobulin would serve as the basis for a serum test for malignancy have not been fulfilled, but this molecule does appear to have prognostic value for patients with lymphoproliferative diseases, particularly multiple myeloma. In addition, recent studies have suggested that β_2_-microglobulin can be used as a prognostic marker in patients infected with human immunodeficiency virus (HIV) [13]. Still, the normal physiological functions of β_2_-microglobulin in vivo have not yet been elucidated. The findings presented here suggest that β_2_-microglobulin serves as an antibacterial agent in HAF.

In this report, we describe the isolation and characterization of an antibacterial protein, β_2_M, from HAF. This protein exhibited potent antibacterial activity against pathogenic microbial strains, and its sequence was identical to that of β_2_-microglobulin.

## Results and Discussion

### Purification of β_2_M protein

**Figure 1:**
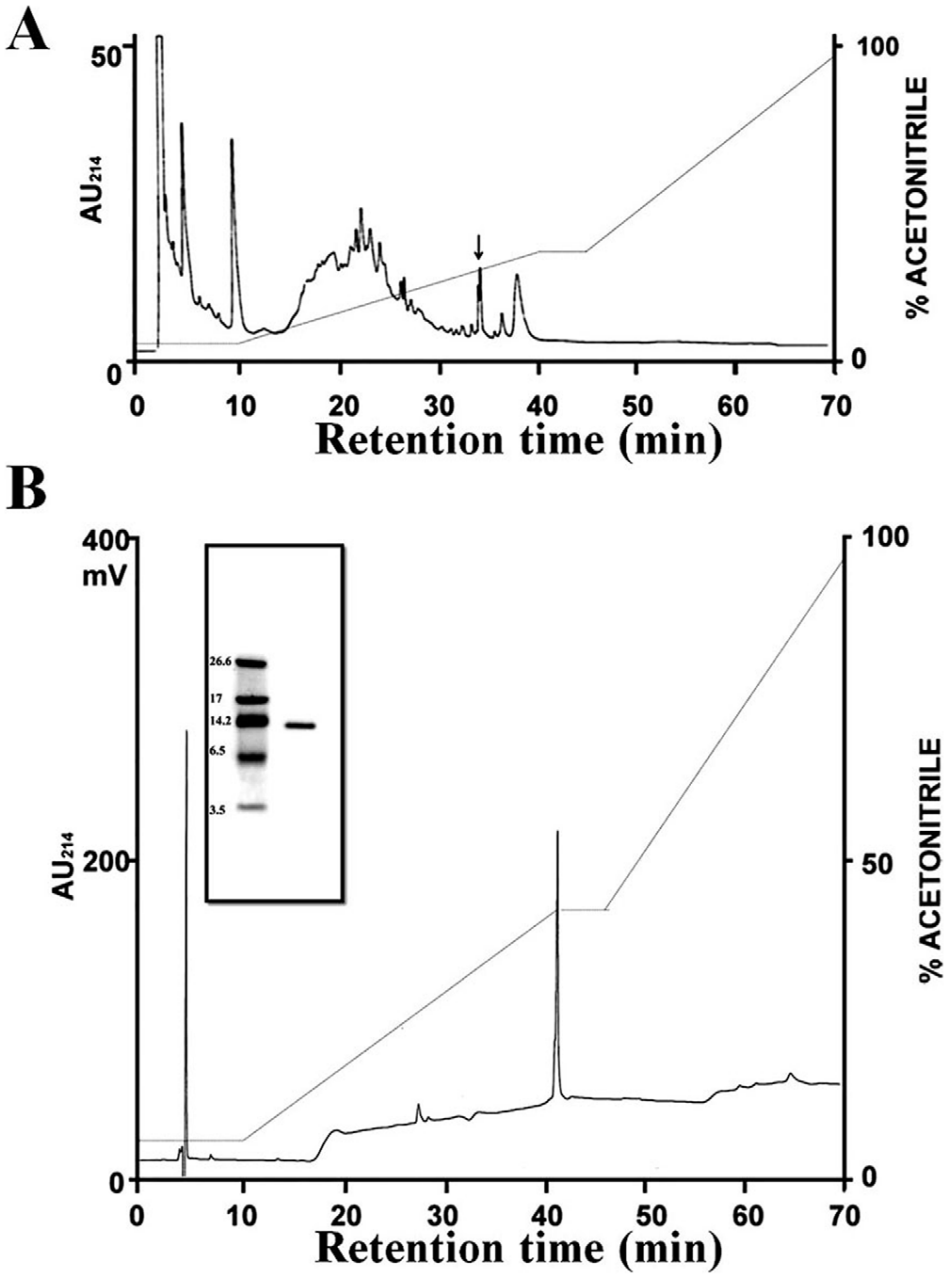
RP-HPLC profiles and Tricine SDS-PAGE analysis of fractions >10 kDa. Following dialysis and ultrafiltration steps, a representative HAF sample was injected into a HPLC system equipped with a Vydac C_18_ column (A). The indicated fraction (arrow) was subjected to a second RP-HPLC (B) and eluted using a gradient of acetonitrile in 0.1% TFA. The purified protein was analyzed by Tricine SDS-PAGE.

Following dialysis and ultrafiltration to remove urine and salts, HAF samples were applied to a RP-HPLC equipped with a C18 column (Fig. 1A). This dialysis process removes salts and small molecules coming from urine but not larger proteins such as β_2_M which has a mass of 11600. Thus the beta2M may come from amniotic fluid but could be contaminated with β_2_M from urine. Because the peak shown in Fig. 1A was found to have potent antibacterial activity against *Listeria monocytogenes*, this fraction was further purified using RP-HPLC with a delayed gradient program (Fig. 1B). The resultant purified protein was homogeneous, and its molecular weight was determined to be about 12 kDa using tricine SDS-PAGE (inset in Fig. 1B). Sequencing revealed that the N-terminal amino acid sequence of β_2_M is NH_2_-Ile-Gln-Arg-Thr-Pro-Lys-Ile-Gln-Val-Tyr-Ser-Arg-His-Pro-Ala-Glu-Asn-Gly-, which was 100% identical to that of β_2_-microglobulin [14]. Moreover, matrix-assisted laser desorption ionization mass spectrometry (MALDI-MS) showed that β_2_M has a molecular mass of 11,631 Da, which is identical to the relative molecular weight calculated from the protein sequence of the peptide (11,631 Da).

### Antibacterial activity of β_2_M against antibiotic-susceptible and -resistant bacteria

**Figure 2:**
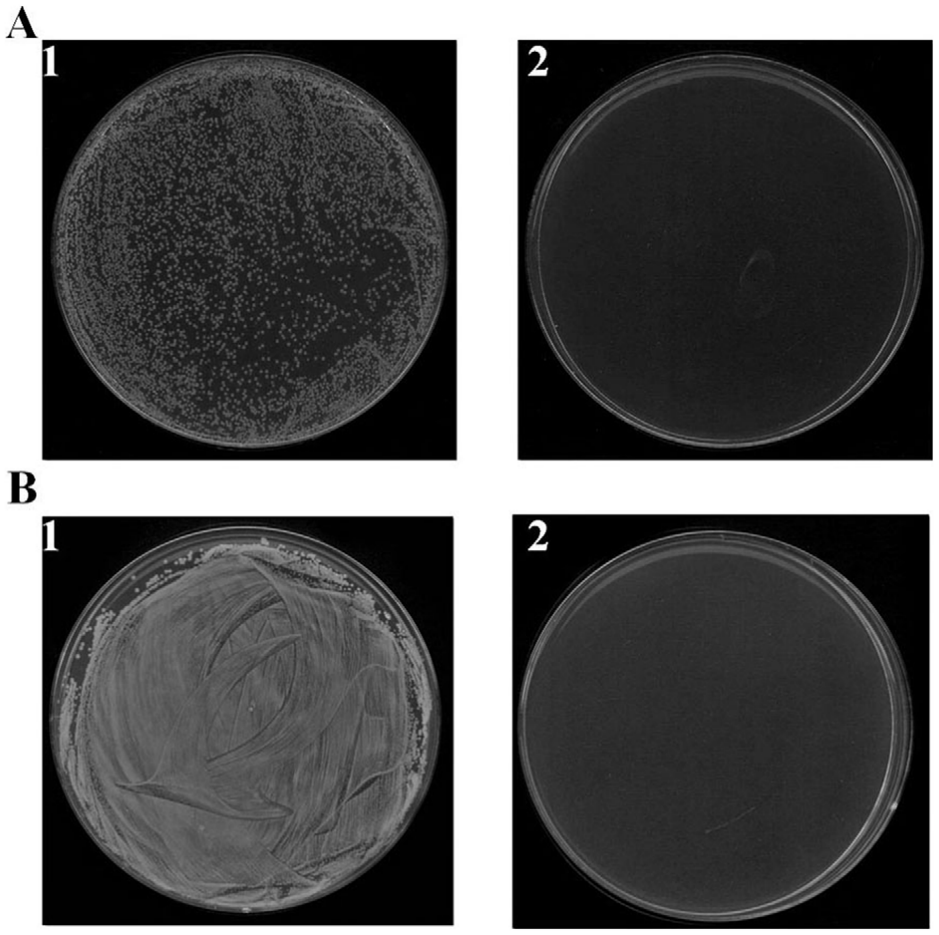
Antibacterial activity of purified β_2_M against *L. monocytogenes* (A) and *E. coli* (B). Shown are representative cultures grown in the absence (1) and presence (2) of β_2_M (2.5 μg).

The antibacterial activity of the purified protein was then assayed against *L. monocytogenes* and *Escherichia coli*, and was found to potently inhibit the growth of both organisms (Fig. 2). In addition, amino acid sequencing revealed this protein to be identical to β_2_-microglobulin, and its molecular mass indicated it to be the mature form of the protein. Although increases in the β_2_-microglobulin concentration in HAF and in adult and fetal serum have been observed under abnormal conditions, the molecular basis of these increases is still not known, nor is the physiological function of β_2_-microglobulin in humans. This study is thus the first to report on the antibacterial properties of β_2_-microglobulin and its expression in amniotic cells in response to pathogenic stimulation.

Determination of the minimum inhibitory concentrations (MICs) revealed that β_2_M more effectively inhibits the growth of antibiotic-susceptible strains (American Type Culture Collection (ACTC) and Korean Collection for Type Cultures (KCTC) strains) than resistant strains (Culture Collection of Antibiotics Resistance Microbes, CCARM strains) (Table 1). To assess the influence of ion salts on the growth-inhibiting activity of β_2_M, antibacterial assays were performed in four buffers, potassium or sodium phosphate (10 mM, pH 7.2), HEPES (10 mM, pH 7.2), and potassium phosphate containing 150 mM NaCl (10 mM, pH 7.2). Under these conditions, β_2_M showed potent antibacterial activity against antibiotic-susceptible bacteria, but not against antibiotic-resistant bacteria. Moreover, β_2_M significantly inhibited growth only in potassium phosphate buffer, indicating that potassium ion salts are essential to its antibacterial activity. Although the solute concentrations in HAF varies, depending on the stage of pregnancy and among individuals, they are reportedly about 130 mEq/L sodium, 4 mEq/L potassium, 110 mEq/L chloride, 3.4 mEq/L calcium and 3 mEq/L phosphorous [15]. Based on these values and the results of the present study, the antibacterial activity of β_2_M we observed in vitro would also be observed in natural HAF. Notably, β_2_M did not inhibit antibiotic-resistant strains. These strains often express a multidrug-resistance (MDR) efflux pump/transporter in their plasma membrane, which alters the membrane potential when activated. Our findings indicate that exposure to β_2_M in potassium ion salt led to alteration of the bacterial membrane potential, which is consistent with the presence of the MDR efflux pump/transporter.

**Table 1:** Minimal inhibitory concentrations of β_2_M against antibiotic-susceptible and resistant pathogens.

Strains	MICs (µM)			
	Buffer I^a^	Buffer II^b^	Buffer III^c^	Buffer IV^d^
*S. aureus* ATCC25923	2.4	>2.4	>2.4	>2.4
*L. monocytogenes* ATCC19115	0.3	>2.4	>2.4	>2.4
*S. epidermidis* KCTC1917	0.6	>2.4	>2.4	>2.4
*E. coli* ATCC25922	0.3	>2.4	>2.4	>2.4
*P. vulgaris* KCTC2433	0.3	>2.4	>2.4	>2.4
*S. typhimurium* KCTC1926	>2.4	>2.4	>2.4	>2.4
*S. aureus* CCARM3114	>10	-	-	-
*S.aureus* CCARM3126	>10	-	-	-
*E. coli* CCARM1229	>10	-	-	-
*E. coli* CARM1238	>10	-	-	-

a Minimum inhibitory concentration in 10 mM potassium phosphate, pH 7.2.

b Minimum inhibitory concentration in 10 mM HEPES, pH 7.2.

c Minimum inhibitory concentration in 10 mM sodium phosphate, pH 7.2.

d Minimum inhibitory concentration in 10 mM potassium phosphate containing 150 mM NaCl, pH 7.2.

ATCC strains were obtained from the American Type Culture Collection. KCTC strains were from the Korean Collection for Type Cultures. CCARM strains were from Seoul Women's University.

### Mechanism of β_2_M antibacterial activity

**Figure 3:**
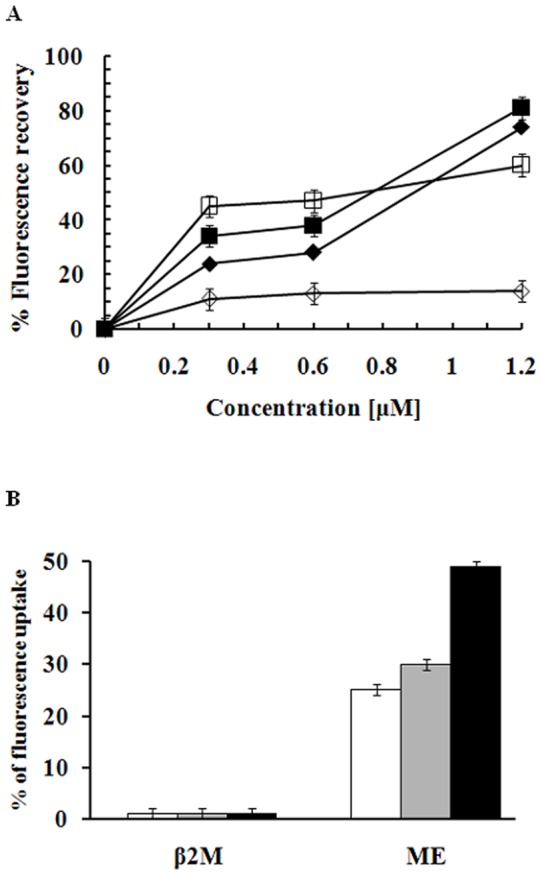
Effect of β_2_M on bacterial membrane potential. Bacteria (*E. coli* ATCC25922, *E. coli* CCARM1229) grown to mid-logarithmic phase were pre-equilibrated for 60 min with disC_3_-5, a voltage-sensitive fluorescent dye. β_2_M was then added to cell suspensions at various concentrations, and changes in fluorescence were recorded. Black, *E. coli* ATCC25922; white, *E. coli* CCARM1229; diamonds, β_2_M; squares, melittin (A). The influx of SYTOX green into *E. coli* ATCC25922 cells after addition of β_2_M and melittin was monitored at an excitation wavelength of 485 nm and an emission wavelength of 520 nm. The maximum increase in uptake was determined using 1% triton X-100 (B).

To investigate the mechanism by which β_2_M inhibits the growth of bacterial cells, we used the membrane potential-sensitive fluorescent probe DiSC_3_-5 to assess the ability of β_2_M to induce membrane depolarization in antibiotic-susceptible (ATCC 25922) and -resistant *E. coli* (CCARM 1229). β_2_M-induced changes in membrane permeability leading to dissipation of the transmembrane potential were monitored by measuring increases in fluorescent emission caused by release of the membrane potential-sensitive dye DiSC3-5. As shown in Fig. 3A, the magnitude of the β_2_M-induced depolarization differed significantly between the two strains: whereas depolarization of *E. coli* CCARM 1229 was minimal, even at a β_2_M concentration of 1.2 μM, maximum depolarization of *E. coli* ATCC 1229 was detected at 0.3 μM β_2_M, which was the MIC for β_2_M with both strains. These results do not confirm that β_2_M interacts with or binds to the cell membrane, but suggest that potassium ion is a unique factor involved in the antibacterial activity of β_2_M. To determine whether β_2_M influences membrane permeability, the influx of SYTOX green into the cytosol of bacterial cells was measured after adding β_2_M to the cells at 1, 5 or 10 times the MIC. When the cell membrane is disrupted or permeabilized by an exogenous agent, SYTOX green dye enters and binds to intracellular nucleic acids, resulting in an increase in the fluorescence [16]. Therefore, β_2_M-induced increases in SYTOX fluorescence, reflecting the binding of the dye to intracellular DNA, were monitored (excitation wavelength: 485 nm, emission wavelength: 520 nm). For example, melittin, a membranolytic peptide, caused a significant influx of SYTOX green. On the other hand, β_2_M did not induce an influx of dye into the cells, even at 10 times the MIC (Fig. 3B). Thus, although the antibacterial action of β_2_M is mediated through dissipation of the membrane potential, β_2_M does not appear to damage the bacterial cell membrane.

### Induced β_2_M mRNA expression in WISH amniotic epithelial cells

**Figure 4:**
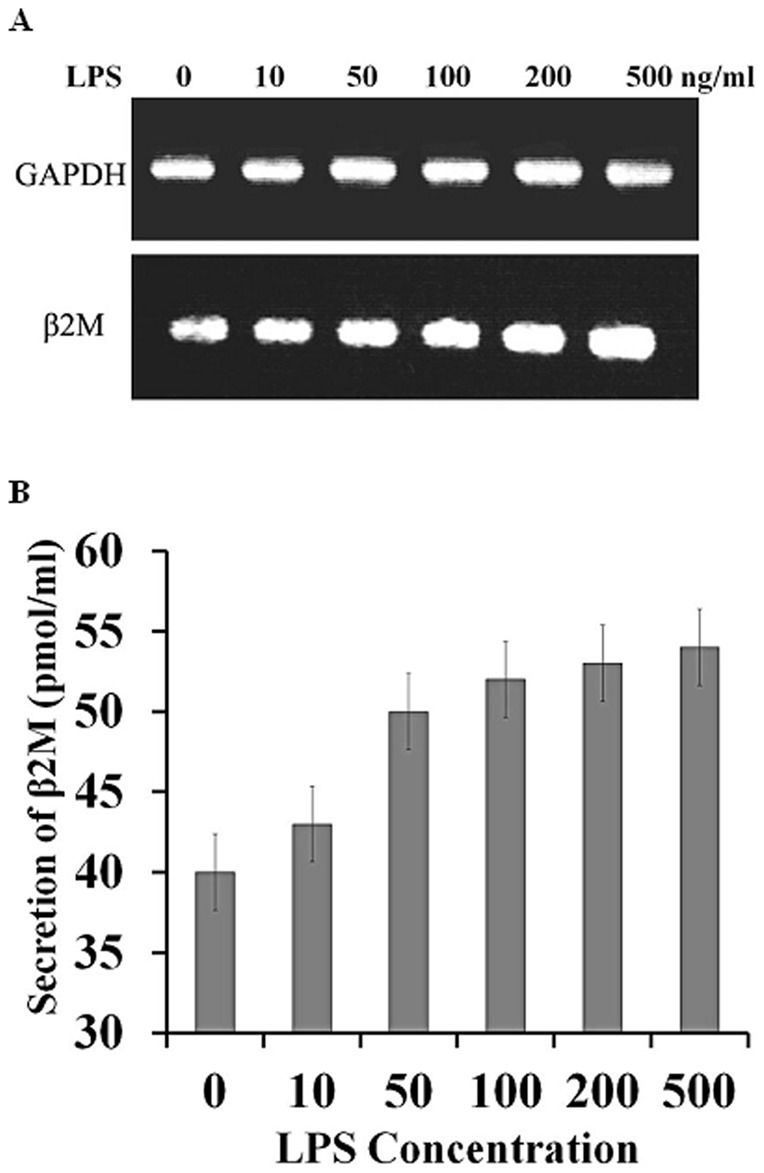
LPS-induced expression and secretion of β_2_M mRNA (A) and protein (B) in cultured WISH cells. WISH amniotic epithelial cells were cultured at a density of 2×10^5^ cells/well for 24 h and then stimulated with LPS (0, 10, 50, 100, 200, 500 ng/ml). GAPDH served as a control in (A). Levels of secreted β_2_M were monitored using an ELISA, based on the 450 nm fluorescence (B).

To determine whether the level of β_2_M expression increases when WISH amniotic epithelial cells are exposed to pathogens, we used RT-PCR to assess its mRNA expression in cells stimulated with bacterial endotoxin LPS. Quantitative analysis following exposure to LPS (0, 10, 50, 100, 200, and 500 ng/ml) revealed a remarkable dose-dependent increase in β_2_M mRNA. By contrast, the housekeeping gene GAPDH was constitutively expressed at the same level, irrespective of the LPS concentration (Fig. 4A). Correspondingly, LPS also elicited concentration-dependent increases in the secretion of β_2_M protein (Fig. 4B). These findings further confirm that β_2_M contributes to the defense against pathogens.

In conclusion, the results of the present study suggest that the antibacterial activity of β_2_-microglobulin in HAF occurs via dissipation of the membrane potential of bacterial pathogens, and that potassium is an important factor in that reaction. In addition, β_2_-microglobulin appears to be upregulated in amniotic cells during bacterial infection.

## Materials and Methods

### Human amniotic fluid

This study was approved by the institutional ethics committee, and all pregnant women provided written informed consent before treatment. Samples of amniotic fluid were obtained from 10 pregnant women in their third trimester. The samples were immediately centrifuged, and the supernatants were sterilized by filtration through a disposable membrane filter (pore size  = 0.2 µm, Millipore), after which they were divided into aliquots and stored at −80°C until needed.

### Purification and mass spectrometry of antibacterial proteins

Samples of HAF were dialyzed against 10 mM ammonium acetate buffer (pH 6.0) to remove urine and salts, after which the dialysates were subjected to ultrafiltration using a membrane with a 10,000 Da molecular weight cut-off. Aliquots of the ultrafiltrates were applied to a RP-C18 column (5 μm, 300 Å, 4.6×250 mm, Vydac, Hesperia, CA) and separated using a 10–60% acetonitrile gradient for 50 min at a flow rate of 1 ml/min. The effluent was monitored by measuring the absorbance at 214 nm, and the peak fractions were assayed for antibacterial activity. The indicated fraction (arrow) was showed most antibacterial activity and only the peak was subjected to a second RP-HPLC. To obtain highly purified homogenous proteins, peak fraction was reanalyzed using a delayed program, an RP-C18 column (5 μm, 300 Å, 2.1×150 mm, Vydac, Hesperia, CA) and a slower flow rate (0.2 ml/min). The purity of the purified peptide was assessed using 16.5% Tricine SDS-PAGE and analytical reversed-phase HPLC [9].

The amino-terminal amino acid sequence of the purified protein was analyzed using automated Edman degradation on a pulse liquid automatic sequencer (Applied Biosystems Inc., model 473A) in the Sequence Centre at the Korea Basic Science Institute (Seoul, Korea). Matrix-assisted laser desorption ionization mass spectrometry (MALDI-MS) was carried out in the linear mode using a Voyager DE RP instrument (Perseptive Biosystems, Framingham, MA) as described by Pouvreau et al. [17].

### Microbial strains

*Streptococcus aureus* (ATCC 259231), *Listeria monocytogenes* (ATCC 19115) and *Escherichia coli* (ATCC 25922) were obtained from the American Type Culture Collection. *Staphylococcus epidermidis* (KCTC 1917), *Proteus vulgaris* (KCTC 2434) and *Salmonella typhimurium* (KCTC 1926) were from the Korean Collection for Type Cultures. *Staph. aureus* CCARM 3114, *Staph. aureus* CCARM 3126, *E. coli* CCARM 1229 and *E. coli* CCARM 1238 were distributed from the Culture Collection of Antibiotic-Resistant Microbes at the Seoul Women's University, Korea.

### Antibacterial activity

The bacteria were grown to mid-logarithmic phase in medium containing (g/l) 10 bactotryptone, 5 yeast extract and 10 NaCl (pH 7.0). The isolated protein was diluted stepwise in 1% bactopeptone medium. The tested organism (final bacterial suspension: 5×10^3^ CFU/ml) suspended in growth medium (100 µl) was then mixed with 100 µl of test peptide solution in a microtiter plate well. Each test solution was examined in triplicate. Microbial growth was determined based on the increase in optical density at 620 nm after incubation for 10 h at 37°C [13].

### Protein quantification and N-terminal sequencing

The protein concentration was determined using the BCA protein assay method [18] with bovine serum albumin as the standard. All protein assays were conducted in triplicate. After homogenous β_2_M was subjected to Tricine-SDS-PAGE, the protein was electroblotted (200 mA for 2 h) onto a polyvinylidene fluoride (PVDF) membrane with a pore size of 0.2 μM. The β_2_M band used for sequencing was cut out of the membrane and air-dried, after which the N-terminal amino acid sequence was determined based on Edman degradation [19] on an amino acid sequencer (Applied Biosystems Inc., Model 473A). Finally, the sequence was compared with sequences in the NCBI database using BLAST.

### Transmembrane potential depolarization

Membrane depolarization in *E. coli* ATCC 25922 and CCARM 1229 cells was assessed using DiSC_3_-5 as described previously [20]. Each treatment protocol was replicated 3 times during the experiment.

### SYTOX green uptake assay

*E. coli* cells were washed and suspended (2×10^7^ cells/ml) in PBS, after which SYTOX green (Molecular probes) was added to a final concentration of 1 μM, and the cells were incubated at 37°C for 15 min with agitation in the dark [21]. Thereafter, β_2_M and melittin were added, and the increase in fluorescence was monitored at an excitation wavelength of 485 nm and an emission wavelength of 520 nm. Each treatment group was assayed in triplicate.

### Expression of β_2_M in amniotic cells

WISH cells, which are a human amniotic epithelial cell line, were obtained from the Korean Cell Line Bank (Seoul, Korea) and grown at 37°C in DMEM supplemented with 10% FBS and 100 U/ml penicillin/streptomycin. The cells were then plated at 2×10^5^ cells/well in a 12-well culture plate in the same media. To evaluate production of β_2_M in response to bacterial stimulation, lipopolysaccharide (LPS) from *E. coli* 255:B5 was added, and the cells were incubated for 24 h [22]. The cells were then washed twice with PBS, and the total RNA was extracted using TRI reagent for later use as a template in RT-PCR. The cDNA products were amplified using the primer pairs, 5′- GGATCCATCCAGCGTACTCCAAAGATTCA-3′ and 5′-CTCGAGTTACATGTCTCGATCCCACTTA-3′ for β_2_M and 5′-CCATCAACGACCCCTTCATTGAC-3′ and 5′-GGATGACCTTGCCCACAGCCTTG-3′ for glyceraldehyde 3-phosphate dehydrogenase (GAPDH). Finally, the PCR-amplified products were electrophoresed on 1% agarose gel [23].

To estimate production of β_2_M protein, amnion epithelial WISH cells were cultured at 2×10^5^ cells/well for 24 h and then stimulated with LPS from *E. coli* 255:B5 (0, 10, 50, 100, 200, 500 ng/ml). Thereafter, the increase in β_2_M was monitored using an ELISA, based on changes in 450 nm fluorescence. Each treatment group was assayed in triplicate.
